# Polymerization-Induced Microphase Separation with Long-Range Order in Melts of Gradient Copolymers

**DOI:** 10.3390/polym12112637

**Published:** 2020-11-10

**Authors:** Alexey A. Gavrilov, Alexander V. Chertovich

**Affiliations:** 1 Faculty of Physics, Lomonosov Moscow State University, 119991 Moscow, Russia; chertov@polly.phys.msu.ru; 2 Semenov Federal Research Center for Chemical Physics, 119991 Moscow, Russia

**Keywords:** gradient copolymers, polymerization, self-assembly, microphase separation, polymerization-induced microphase separation, computer simulations, dissipative particle dynamics

## Abstract

In this work, we studied the question of whether it is possible to develop a one-step approach for the creation of microphase-separated materials with long-range order with the help of spontaneous gradient copolymers, i.e., formed during controlled copolymerization solely due to the large difference in the reactivity ratios. To that end, we studied the polymerization-induced microphase separation in bulk on the example of a monomer pair with realistic parameters based on styrene (S) and vinylpirrolydone (VP) by means of computer simulation. We showed that for experimentally reasonable chain lengths, the structures with long-range order start to appear at the conversion degree as low as 76%; a full phase diagram in coordinates (fraction of VP—conversion degree) was constructed. Rather rich phase behavior was obtained; moreover, at some VP fractions, order–order transitions were observed. Finally, we studied how the conversion degree at which the order–disorder transition occurs changes upon varying the maximum average chain length in the system.

## 1. Introduction

Gradient copolymers are a special class of macromolecules in which the chain composition smoothly changes along the chain [1,2]. This feature of the monomer sequence results in unique interfacial [3,4,5], mechanical [1], thermal [6,7], and self-assembling properties [8,9,10,11,12]. The necessary conditions for the synthesis of gradient copolymers are fast initiation, simultaneous growth of all chains, and the absence of termination and side reactions [13,14,15]; only under such conditions will the macromolecules turn out to be similar. The recent advances in controlled polymerization make it possible to significantly expand the class of macromolecules that can be synthesized in the form of gradient copolymers [13,16,17,18,19,20]. Living anionic polymerization is used for the synthesis of gradient copolymers as well [21,22,23,24].

Gradient copolymers can be divided into two classes based on the synthesis approach: forced and spontaneous [2,13,14,15]. The gradient sequence structure of the forced gradient copolymers is realized by the specific synthetic route, namely the presence of continuous feeding of one monomer type into the reaction media throughout the polymerization. On the other hand, spontaneous gradient copolymers are produced spontaneously due to the feed composition drift that occurs naturally during the reaction. From this circumstance, the main requirement for the synthesis of such gradient copolymers arises; in order to achieve a significant change in the monomer composition along the polymer chains, the difference in reactivity ratios (r_1_ and r_2_) must be rather substantial. Both these approaches have their own advantages and disadvantages; while the former one has much fewer restrictions on the monomer pairs suitable for the copolymerization as well as precise control over the resulting copolymer profiles, it requires a more sophisticated synthetic procedure due to the necessity to change the monomer feed. The latter one, on the other hand, is applicable to a fewer number of monomer pairs, but the synthetic procedure is more straightforward.

Recently, a novel approach for the creation of nanostructured materials, which can be called by the collective name polymerization-induced structuring, has emerged. Essentially, the reported applications of this approach can be divided into two big categories: structuring in solvent and in bulk. The former one is widely known as polymerization-induced self assembly (PISA), which is recognized as an efficient technique to produce block copolymer nanoparticles and has been studied rather extensively [25,26,27]. In the typical PISA approach, a macromolecular initiator is prepared; then, in the second step, it is placed in a selective solvent together with the monomer of the second block. During polymerization, the insoluble second block grows, inducing self-assembly to form micelles [27]. The paradigm of PISA was extended toward creating a one-step approach, and in recent works [28,29] the micelle formation during the gradient copolymer synthesis was reported for both “forced” [29] and “spontaneous” [28] gradient copolymers. The structuring in bulk, which is often referred to as polymerization-induced phase separation (PIPS) or microphase separation (PIMS) [30,31,32,33], has been studied significantly less than PISA; moreover, the majority of works on this topic deal with structures obtained through kinetic arrest or cross-linking [31]. To the best of our knowledge, PIMS resulting in structures with long-range order has almost not been studied (there are examples of graft polymerization-induced [34,35] formation of such structures), and gradient copolymers, given the recent advances in PISA [28,29], seem to be an excellent candidate for further investigation in this area.

The main goal of this work is to answer the question of whether it is possible to develop a one-pot paradigm (i.e., without any additional polymerization and equilibration steps) for the creation of microphase-separated materials with long-range order with the help of spontaneous gradient copolymers. To that end, we studied the polymerization-induced microphase separation during copolymerization in bulk on the example of a monomer pair with realistic parameters based on styrene (S) and vinylpirrolydone (VP) by means of computer simulation. It is worth noting that the monomer pair of VP and S was chosen simply as one of the examples of such systems [1,36,37], as the gradient structure of the copolymers is formed during controlled copolymerization without any additional conditions simply due to the large difference in the reactivity ratios. The main conclusions of this work should be valid for other similar monomer pairs as well.

## 2. Method and Model

For the simulations, we used the dissipative particle dynamics (DPD) method [38]. The detailed description of the used method as well as parameters used are presented in Supplementary Information. 

In order to simulate the controlled polymerization process, we used the scheme described in detail in [39]. In this procedure, a nondormant growing chain end of the type α forms a new bond with a free monomer of the type β located within the distance *R*_chem_ (in our case, *R*_chem_ = *R*_c_ = 1) from the chain end with the probability *p*_αβ_. The probability matrix defines the reactivity ratios in the case of copolymerization; the reactivity ratios are equal to *r*_α_ = *p*_αβ_ /*p*_αα_. The detailed description of the used approach is presented in the Supplementary Information. While the scheme described in [39] allows for simulation of the dormant chain ends as well as the chain termination, in this work, we use the idealized procedure, taking only initiation and chain propagation into account. This circumstance will lead to somewhat low dispersity; however, we feel that, given the overall complexity of the studied system, it is correct to investigate the effect of the nonideality of the copolymerization reaction separately.

In this work, we simulate the self-assembly during the controlled radical copolymerization of model S and VP monomers in bulk. The synthesis of gradient copolymers from this monomer pair by TEMPO-mediated copolymerization was reported in [1]. This pair has the reactivity ratios of *r*_s_ = 17.39 and *r*_vp_ = 0.058 [40]. Such values suggest that (if the chain termination and chain transfer processes are suppressed in the system, i.e., controlled polymerization is realized) during the early stages of copolymerization, styrene monomers will join the chains predominantly, and, after its exhaustion, pure vinylpyrrolidone blocks will grow. The initial part of the growing chains, however, will contain some amount of vinylpyrrolidone, so the resulting gradient copolymer chains are not symmetric in respect to their centers.

In order to estimate the incompatibility parameter χ between the monomers, we used the Hildebrand solubility parameters δ and calculated χ using the well-known formula: $\chi =\frac{v_{\mathrm{mol}}^{\mathrm{av}}}{\mathrm{RT}}{\left(\delta_{S}-\delta_{\mathrm{VP}}\right)}^{2}$, where $v_{mol}^{av}=\frac{v_{mol}^{styrene}+v_{mol}^{VP}}{2}$ is the average molar volume of the species and *R* is the universal gas constant. Using the values δ_S_ ≈ 19 MPa^1/2^ [41] and δ_VP_ = 24.3 MPa^1/2^ [42], we obtain the χ value of 1.05 at room temperature, indicating that the chosen monomers are rather incompatible. Given the inexact nature of such a simple method of the determination of χ-parameter, we, for simplicity, will assume that χ_VP-S_=1 (unless otherwise stated). It is worth noting that such an χ-value indicates that the chosen monomer pair forms a stable mixture (i.e., they are miscible) for any monomer fractions, and the segregation occurs only after polymer chains of certain length (see below) start to grow. 

The initial monomer mixture is characterized by the fraction of VP in it, *f*_vp_. Since we study bulk copolymerization, the system contains three types of beads: initiator and two types of monomers, S and VP. The fraction of initiator is directly related to the maximum (at 100% conversion) average chain length *N*_max_; indeed, when all the bonds are formed, the average chain length is equal to just *n_monomer_/n_initiator_* + 1 (where *n_monomer_* and *n_initiator_* are the total monomer and initiator number density, respectively), i.e., *N*_max_ is equal to the number of monomers per one initiator in the initial mixture plus initiator itself. In the majority of our simulations, we used *N*_max_ = 80; this value is reasonable from the experimental point of view, but it still allows one to perform investigations in simulation boxes of adequate sizes. It is worth noting that the results of the present work are not specific for the chosen *N*_max_, and the main conclusions should be valid for other *N*_max_ values as well. In order to study the generalized case, we assumed that the initiator has χ = 0 with both monomer types, and the initiation probability for the monomers is also the same. The simulation box was cubic with periodic boundary conditions in all three directions; its size was equal to 50^3^ (375,000 beads), unless stated otherwise.

## 3. Results and Discussion

**Figure 1:**
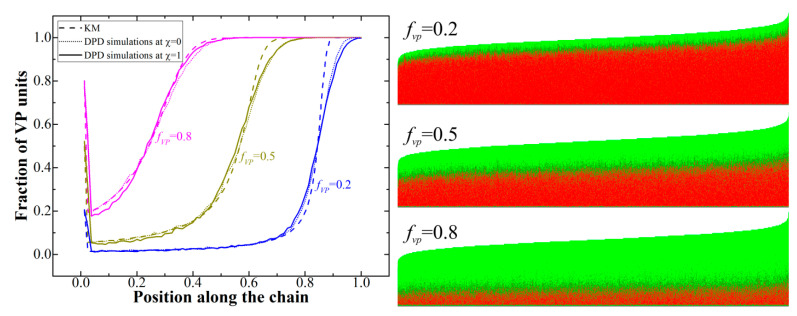
**Left**: Gradient copolymers profiles at conversion degree 100% obtained from kinetic model and dissipative particle dynamics (DPD) simulations; **Right**: graphical representation of all the chains in the system with red being S monomer units and green being VP monomer units.

First of all, we studied the profiles of the copolymers that can be observed for the chosen monomer pair at different values of *f*_vp_. To that end, we applied the Monte Carlo model in the sequence space [39]; we will call it the kinetic model (KM) in what follows (see the Supporting Information for more details). In short, this approach does not take into account the distribution of the monomers and cross-linker in space, but rather calculates the copolymerized sequence using the probabilistic approach based on the remaining concentration of the monomers and their reactivity ratios *r*. The results obtained using the kinetic model were compared to the results of DPD simulations, for which two different cases were considered: an ideal one with χ = 0 as well as the case of nonzero χ (χ = 1, see above). Figure 1 (left) depicts the obtained copolymers profiles; Figure S2 (see the Supplementary Information) additionally depicts the conversions of S and VP monomers depending on the overall reaction conversion.

We can see that the resulting copolymers are in fact gradient formed spontaneously during the copolymerization due to the fast consumption of the S monomer at the early reaction stages (Figure S2); they can be divided into two regions (blocks), enriched with styrene and pure VP. The spike observed at small chain length is related to that fact that the initiation probability is the same for both monomers, and the fraction of polymer chains with the first unit being, say, VP, is simply equal to the fraction of VP in the initial monomer mixture. As it was expected, the results obtained from the DPD simulations at χ = 0 and KM are in good agreement; the differences observed in the transition region (which is smoother for the DPD simulation results) are most probably related to the chain polydispersity (even though the obtained dispersity value Ð of 1.012–1.013 is rather small, see the graphical chain representations in Figure 1 (right)) as well as the simplified growth process in the KM where all the chains grow simultaneously. At χ = 1, we see slight deviations from the KM data in the region of the styrene block; this can explained by the presence of the bootstrap effect [43], when the growing polymer chain due to the presence of the incompatibility between monomers of different types controls its own environment.

Indeed, we found that, at the conversion degree of 4% and f_VP_ = 0.5, the observable values of r obtained from the analysis of the chain sequences obtained from the DPD simulations at χ = 1 are ${r\prime }_{S}$ = 20.25 ± 0.61 and ${r\prime }_{VP}$ = 0.065 ± 0.004 (averaged over eight runs), while at χ = 0, they were equal to 17.34 ± 0.32 and 0.059 ± 0.003; the latter two are, as expected, in good agreement with the input values. The observable values r’_S_ = p’_S-S_/p’_S-VP_ and r’_VP_ = p’_VP-VP_/p’_VP-S_ were calculated from the real probabilities to find two consequential S-units, two consequential VP-units, S-unit after VP-unit, and vice-versa (i.e., p’_S-S_, p’_VP-VP_, p’_S-VP_, and p’_VP-S_) by scanning along the chains. As we can see, both values of r increase for the high χ-value, which is in agreement with the previous study [39]. However, from Figure 1, we can see that the nonzero χ has only a subtle effect on the copolymer profiles. This is essential, given the fact that the experimental values of r obtained in the work [44] from the solution copolymerization data are also most likely inherently different from the true values (i.e., those somehow uninfluenced by any other interactions) due to the incompatibility between the species (monomers and solvent). In our copolymerization model, however, we need to use the true values of r, as the reaction probabilities depend only on the types of reacting species. Nevertheless, while it seems possible to adjust the input values of r for the simulation to take into account the interactions present in the experimental system used for the determination of the copolymerization ratios, the effect of such a correction on the phase behavior should be minor as the gradient profiles do not seem to change due to the introduction of nonzero χ.

**Figure 2:**
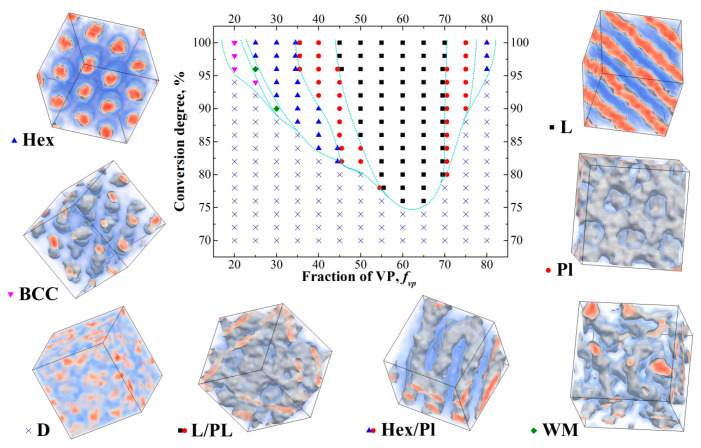
Obtained phase diagram during controlled copolymerization of S and VP in bulk. The observed structures are: D—disordered, L—lamellar, PL—perforated lamellae, Hex—hexagonally packed cylinders, S—body-centered cubic structure, WM [45]—wormlike micelles. Two types of transitional structures were observed: Hex/Pl and L/Pl. The first one is hexagonally packed cylinders, some of which are connected to each other by rare bridges; the second one is lamellae with some number of perforations, which appear to have no order within every layer.

It should be noted that in controlled radical polymerization it is usually hard to reach full conversion; therefore, it seems crucial to determine whether the structure formation can occur at incomplete conversions. Figure 2 depicts the obtained phase diagram in the coordinates *f*_vp_-conversion degree (we remind that the Flory–Huggins parameter χ_VP-S_ between S and VP beads is equal to 1).

In order to construct the phase diagram, we chose small enough reaction probabilities so that the system reaches 90% conversion in about 55 × 10^6^ steps. After that conversion was reached, all the reaction probabilities were continuously adjusted to ensure that the number of new bonds in the system formed every 40,000 steps was kept at the same level; this is necessary in order to ensure that the system reaches high conversions in a reasonable time. The adapted procedure allowed us to reach 100% conversion in (100–110) × 10^6^ steps; such slow reaction is necessary to allow the system to form equilibrium structure (or at least structure close to being equilibrium) at any given conversion degree. Overall, the slow growth of the chains is, on average, characteristic for controlled radical polymerization as the growing ends remain in the dormant state most of the time. In order to define the positions of the order–disorder transitions (ODT) more precisely, we performed additional 30 × 10^6^ steps-long runs at all conversion degrees around the initially defined ODT conversion degrees with the reaction turned off.

We can see from Figure 2 that even at *N*_max_ = 80, the structures with long-range order start to appear at the conversion degree of 76%; at 86% conversion, the ordered structures are obtained in a rather wide range of the *f*_vp_ values from 0.35 to 0.7. We can also see that the system overall demonstrates rather rich phase behavior; we observed all the major phases typical for diblock-copolymers as well as gradient copolymers [9]. We can also see that the resulting diagram is highly asymmetric, which can be explained by two reasons. The first reason is that the blocks forming different domains are not symmetrical. Moreover, the fraction of VP in the S-block varies as the value of *f*_vp_ changes (Figure 1), which complicates the system behavior. The second reason is related to the fact that at intermediate conversions copolymers have more symmetric blocks at large values of *f*_vp_. A simple example of that can be seen if we look at the copolymer profile at *f*_vp_ = 0.8 (Figure 1) and consider the case of the conversion degree of around 50% when the VP block is still rather short. This probably explains why the structures with long-range order are observed at the lowest conversion degree of 76% at *f*_vp_ = 0.6–0.65 and not observed at the same conversion degree for *f*_vp_ = 0.55. Additionally, we observed order–order transitions as the conversion increases at some values of *f*_vp_. This result is explained by the fact that the ratio of the lengths of the S and VP blocks changes as the chain grows.

For example, at the conversion degree of 90% at *f*_vp_ = 0.5, the length of the VP block is not large enough to form lamellae; therefore, perforated lamellae are formed instead. In this case, the nonpolymerized free VP monomer plays the role of selective solvent; however, due to the presence of monomer units of VP in the S block as well as the high translational entropy, the residual VP monomers are not located entirely in the phase formed by the vinylpyrrodidone block, but distributed more evenly in the system. This means that as the reaction goes in the system, the volume of the phases formed by the S and VP blocks changes. 

**Figure 3:**
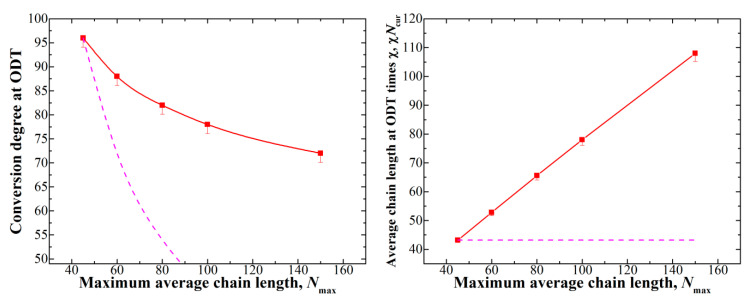
**Left**: dependence of the conversion degree at which the ODT occurs depending on the maximum average chain length *N*_max_; **Right**: dependence of the χ*N*_cur_ of the ODT on *N*_max_. For *N*_max_ = 100, the box size of 60^3^ was used, while for *N*_max_ = 150, the box size was equal to 80^3^. The dashed lines show the behavior if χ*N*_cur_ at the ODT was constant and are given as a guide for the eye.

The position of the conversion transition points will shift upon changing the amount of initiator (i.e., *N*_max_) in the system. Indeed, using longer chains increases χ*N*_cur_ at any given conversion. The effect, however, should be complex due to the fact that as the conversion changes, the ratio of the block lengths as well as the amount of free monomer left in the system change simultaneously. Indeed, at *f*_vp_ = 0.5 and *N*_max_ = 80 (see Figure 2), the ODT occurs at the conversion of 82%; if we consider the same system but with doubled maximum average chain length *N*_max_ = 160, the same χ*N*_cur_ value of ≈66 would technically be reached at the conversion of 41%, but, as we can see from Figure 1, at such conversion, the VP block has not even started forming. The amount of free nonpolymerized monomer is also quite large for such a low conversion degree. The resulting dependence of the ODT conversion degree on *N*_max_ is presented in Figure 3 (left). 

We see that the ODT conversion degree does not change significantly at large values of N_max_ (which, once again, correspond to low initiator amounts); in order to provide a more descriptive characteristic, we calculated the value of χN_cur_ (i.e., average chain length times χ) at the ODT (see Figure 3 (right)). This dependence clearly confirms our considerations described above, as we see that the χ*N*_cur_ value necessary for the formation of ordered structures grows as the *N*_max_ increases. We can assume that, since the ODT cannot occur before the VP block starts to grow, even in systems with very high *N*_max_, the conversion degree at which ODT occurs stays somewhere between 50% and 60% for *f*_vp_ = 0.5 (around these conversions, the VP block appears; see Figure 1).

Finally, we should note an interesting feature of the studied systems. Since the ODT for most *f*_vp_ occurs not at 100% conversion, the remaining unpolymerized monomer acts as solvent, which may help to reach the equilibrium state and avoid kinetic trapping; studying this circumstance is out of the scope of the present letter, but can be an interesting topic for a future work.

## 4. Conclusions

Summarizing, in this work we studied polymerization-induced microphase separation in melts of copolymers formed by model monomers based on styrene and vinylpyrrolidone by means of computer simulations. Due to the large difference in the reactivity ratios, controlled radical copolymerization of these monomers leads to the formation of gradient copolymers with two well distinguished regions (pseudo-blocks): S-enriched and pure VP.

Due to the rather significant incompatibility between the monomers, which was assessed using the solubility parameters, the resulting melt is capable of microphase separation. We showed that the presence of incompatibility between monomers of different types changes the observable reactivity ratios; however, the resulting changes in the copolymer profiles seem to be rather subtle, and it is unlikely that they will change the phase behavior of the system. For the case of the experimentally reasonable maximum average chain length of *N*_max_ = 80, we studied the phase diagram in the coordinates (VP content in the monomer mixture *f*_vp_—conversion degree); we showed that the structures with long-range order start to appear at the conversion degree as low as 76%, which is essential, given that it is usually challenging to reach high conversions during experimental synthesis. We observed order–order transitions as the conversion increased at some values of *f*_vp_, which we explained by the fact that the ratio of the lengths of the S and VP blocks changes as the chains grow. Finally, we studied how the conversion degree at which the ODT occurs shifts upon varying *N*_max_ and showed that the ODT conversion degree does not change significantly at large values of *N*_max_, since it cannot be lower than the conversion degree at which the VP block starts to grow.

We hope that our studies will facilitate the investigation of one-pot strategies to obtain microphase-separated structures in such spontaneous gradient copolymers, which seem to be promising candidates for various applications.

## Supplementary Materials

The following are available online at https://www.mdpi.com/2073-4360/12/11/2637/s1: Description of the simulation method and the kinetic model (KM); Dependences of the monomers conversions on the overall reaction conversion.
