# Supplementary material for: Polymerization-Induced Microphase Separation with Long-Range Order in Melts of Gradient Copolymers
Source: Polymers (Basel). 2020 Nov 10;12(11):2637. doi: 10.3390/polym12112637 (PMC7696285; doi:10.3390/polym12112637)
Supplement: Supplementary file 1 [file polymers-12-02637-s001.pdf]

Supplementary Information for:

Polymerization-induced microphase separation with long-range order in melts of gradient copolymers

Alexey A. Gavrilov[§], Alexander V. Chertovich[§#]
[§]*Faculty of Physics, Lomonosov Moscow State University, 119991 Moscow, Russia*
[#]*Semenov Federal Research Center for Chemical Physics, 119991 Moscow, Russia*

DPD Method and parameters

DPD is a well known simulation technique which has been utilized to simulate properties of a wide range of polymeric systems. Macromolecules are represented in terms of the bead-and-spring model, with beads interacting by a conservative force (repulsion) $\boldsymbol{F}_{ij}^{c}$, a bond stretching force (only for connected beads) $\boldsymbol{F}_{ij}^{b}$, a dissipative force (friction) $\boldsymbol{F}_{ij}^{d}$, and a random force (heat generator) $\boldsymbol{F}_{ij}^{r}$. The total force is given by:

$$\boldsymbol{F}_i = \sum_{i \neq j} \left(\boldsymbol{F}_{ij}^{c} + \boldsymbol{F}_{ij}^{b} + \boldsymbol{F}_{ij}^{d} + \boldsymbol{F}_{ij}^{r} \right) \quad (1)$$

The soft core repulsion between *i*- and *j*-th beads is equal to:

$$\boldsymbol{F}_{ij}^{c} = \begin{cases} a_{\alpha\beta}(1 - r_{ij} / R_c)\boldsymbol{r}_{ij} / r_{ij}, & r_{ij} \le R_c \\ 0, & r_{ij} > R_c \end{cases}, \quad (2)$$

where r_{ij} is the vector between *i*-th and *j*-th bead, $a_{\alpha\beta}$ is the repulsion parameter if the particle *i* has the type α and the particle *j* has the type β and R_c is the cutoff distance. R_c is basically a free parameter depending on the volume of real atoms each bead represents[1]; R_c is usually taken as the length scale, i.e. R_c=1. All beads have the same size in such formulation.

If two beads (*i* and *j*) are connected by a bond, there is also a simple spring force acting on them:

$$\boldsymbol{F}_{ij}^{b} = -K(r_{ij} - l_0)\frac{\boldsymbol{r}_{ij}}{r_{ij}}, \quad (3)$$

where *K* is the bond stiffness and l_0 is the equilibrium bond length. In our simulations we used the following set of parameters: $a_{\alpha\alpha}$=25, *K*=4, l_0=0. More detailed description and parameters discussion of the standard DPD scheme can be found elsewhere [1].

It was shown[1] that the Flory-Huggins interaction parameter χ has a linear dependence on the difference of the DPD repulsion parameters $\Delta a = a_{\alpha\beta} - a_{\alpha\alpha}$; for simple liquids with $a_{\alpha\alpha}$=25 and the number density ρ=3 the relation has the form $\chi=(0.286\pm0.002)\Delta a$. The exact coefficient can vary depending on the type of studied species as well as the parameters such as $a_{\alpha\alpha}$, K and l_0[1–3]. Since the monomer sequence of gradient copolymers is rather complex, the coefficient is rather hard to define since it may slightly vary along the chain; however, for ρ=3 the reported values [1–3] vary not very significantly and lie between ~0.25 and ~0.3 for various species type and the parameters used. Therefore, for simplicity in this work we will use the relation $\chi \approx 0.3\Delta a$.

Reaction scheme

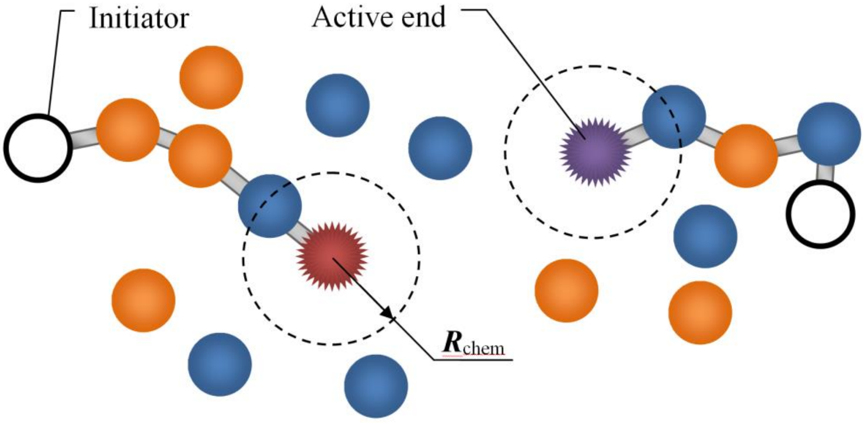

Fig. S1. Schematic illustration of the reaction scheme

In our work we used the reaction scheme described in detail in the work [4]. To mimic the reaction process we use rather Monte Carlo scheme, and the reaction procedure runs after each τ_0 DPD steps. It is worth noting that neither the presence of dormant chain ends nor the side reactions (termination and chain transfer) are considered in our work; the influence of those factors will be studied in the future works. The reaction procedure consists of the following stages (see Fig. S1):

1) A growing chain end or initiator bead i is selected at random;

2) The list of all free monomer beads located closer than the reaction radius R_{chem} from the bead i is created. The closest bead j is determined, and a bond between the beads i and j is created with the probability p_{ij}. If the bond is not created, the procedure is repeated with the next closest bead from the list until a bond is created or there are no more unchecked monomers.

These stages are repeated M times where M is the total number of the chain ends plus initiators, so that on average every growing chain is checked once every time we run the reaction procedure. The reaction radius R_{chem} was chosen to be equal to 1.0, i.e. to the interaction potential cutoff distance R_c.

Kinetic model

In addition to DPD simulations, we used a simple kinetic Monte Carlo model in the sequence space [4]. In this model we additionally took into account that the reaction volume is finite and the monomer volume fractions change during the copolymerization process. The first monomer in the chain is chosen according to the current monomer volume fractions φ_S and φ_{VP} (i.e. the initiation probability is the same for both monomer types similar to the DPD simulations); the propagation probabilities depend on r_S and r_{VP} as follows: $p^{MC}_{S\text{-}S} = \dfrac{r_S\varphi_S}{1-\varphi_S + r_S\varphi_S}$, $p^{MC}_{S\text{-}VP} = \dfrac{1-\varphi_S}{1-\varphi_S + r_S\varphi_S}$, $p^{MC}_{VP\text{-}VP} = \dfrac{r_{VP}\varphi_{VP}}{1-\varphi_{VP} + r_{VP}\varphi_{VP}}$, $p^{MC}_{VP\text{-}S} = \dfrac{1-\varphi_{VP}}{1-\varphi_{VP} + r_{VP}\varphi_{VP}}$, thus representing a simple markovian process. The obtained sequences all have the same length of the corresponding N_{max}, i.e., the chains are monodisperse.

Monomer conversion vs. overall conversion

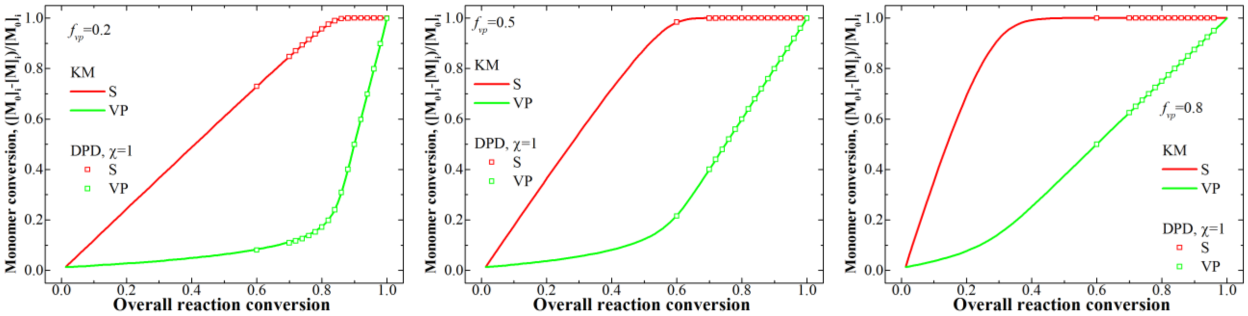

Fig. S2. Dependences of the S and VP monomers conversions on the overall reaction conversion for f_{VP}=0.2, 0.5 and 0.8 (from left to right) obtained using kinetic model (KM) and DPD simulations at χ=1.

References

1. Groot, R.D.; Warren, P.B. Dissipative particle dynamics: Bridging the gap between atomistic and mesoscopic simulation. *J. Chem. Phys.* **1997**, *107*, 4423–4435.
2. Rumyantsev, A.M.; Gavrilov, A.A.; Kramarenko, E.Y. Electrostatically Stabilized Microphase Separation in Blends of Oppositely Charged Polyelectrolytes. *Macromolecules* **2019**, *52*, 7167–7174.
3. Gavrilov, A.A.; Kos, P.I.; Chertovich, A. V. Simulation of phase behavior and mechanical properties of ideal interpenetrating networks. *Polym. Sci. Ser. A* **2016**, *58*, 916–924.
4. Gavrilov, A.A.; Chertovich, A. V. Copolymerization of Partly Incompatible Monomers: An Insight from Computer Simulations. *Macromolecules* **2017**, *50*, 4677–4685.
